# Longitudinal periapical radiographic evaluation of apexification, vital pulpotomy, and revascularization in immature permanent teeth: a retrospective comparative study

**DOI:** 10.3389/fbioe.2026.1792713

**Published:** 2026-04-24

**Authors:** Xiaona Sun, Kailing Zhu

**Affiliations:** 1 Preventive Dentistry, Wuxi Stomatological Hospital, Wuxi, Jiangsu, China; 2 Department of Pediatric Dentistry 1, Wuxi Stomatological Hospital, Wuxi, Jiangsu, China

**Keywords:** a retrospective study, apexification, imaging evaluation, revascularization, vital pulpotomy, young permanent teeth

## Abstract

**Background:**

Immature permanent teeth pose therapeutic challenges due to immature root development; primary approaches include apexification, vital pulpotomy and revascularization, with few systematic imaging studies on their efficacy.

**Aim:**

This study systematically compares the radiographic outcomes of apexification, vital pulpotomy, and pulp revascularization in managing immature permanent teeth, assessing root development and periapical healing to inform clinical decision-making.

**Methods:**

This retrospective cohort analysis employed propensity score matching (PSM) to evaluate treatments for immature permanent teeth. Patients treated between January 2022 and December 2024 were categorized into three groups: apexification (n = 51), vital pulpotomy (n = 50), and revascularization (n = 51) after 1:1 nearest-neighbor matching. Primary outcomes were resolution of clinical signs and symptoms and periapical lesion healing; secondary outcomes were root development (increase in root length and canal wall thickness) and apical closure. Outcomes were assessed radiographically using pre- and postoperative periapical radiographs and cone-beam computed tomography (CBCT) at 6- and 12-month follow-ups.

**Results:**

After PSM, baseline characteristics were balanced (*P* > 0.05). At the 12-month follow-up, the revascularization group demonstrated significantly greater increases in both root length (1.8 ± 0.4 mm) and canal wall thickness (0.35 ± 0.05 mm) compared to the other interventions (*P* < 0.05). While apical closure rates were comparable between revascularization (88.2%) and vital pulpotomy (82.0%), both significantly outperformed apexification (56.9%, *P* < 0.01). Similarly, treatment success rates were higher in revascularization (88.5%) and vital pulpotomy (92.0%) than in apexification (76.5%, *P* < 0.05). Revascularization achieved superior periapical lesion healing (95.0%) versus both apexification (76.9%) and vital pulpotomy (72.0%) (*P* < 0.05). Complication rates showed no significant intergroup differences (*P* > 0.05).

**Conclusion:**

Revascularization demonstrated superior efficacy in promoting root maturation, followed by vital pulpotomy and apexification. It is recommended to choose a personalized treatment plan according to the condition of the affected teeth.

## 1 Introduction

Root development of young permanent teeth is a complex and critical physiological process that usually takes years to complete after tooth eruption. During this period, the root is not fully formed, the apical foramen is open, and the root canal wall is thin and prone to fracture. Therefore, the tooth is susceptible to dental pulp infection and necrosis due to caries, trauma, and other factors, leading to the cessation of root development. This not only affects the stability and function of the teeth themselves, but also poses a serious threat to the patient’s occlusal development and long-term oral health (Zhou et al., 2022; Lee et al., 2023; Hugar et al., 2022). Therefore, how to effectively treat such affected teeth and promote their root development has always been the core challenge and research hotspot in the field of pediatric dentistry and endodontics.

In order to meet this challenge, clinical researchers have developed a variety of treatment strategies. Traditional apexification achieves the retention of affected teeth by forming a hard tissue barrier through drug induction of the root tip, which lays the foundation of treatment in this field (Agrafioti et al., 2017). Subsequently, the application of vital pulpotomy has significantly improved the success rate and efficiency of apical closure by retaining healthy root pulp tissue and cleverly utilizing the development potential of the dental pulp itself (Zhang L. et al., 2025). In recent years, remarkable progress has been made in revascularization based on the concept of tissue regeneration. This technology aims to induce the regeneration of dynamic pulp-like tissue in the root canal, which not only pursues apical closure, but also aims to achieve real continuous development of root length and thickness, which represents the future development direction of this field (Li et al., 2024; Brizuela et al., 2022). Extensive research has established the efficacy and safety of these three therapeutic approaches. For example, a number of case reports and series of studies have shown the great potential of revascularization in promoting root development, and long-term clinical observation has affirmed the excellent value of vital pulpotomy in some cases of pulp survival (Chai et al., 2023; Feng et al., 2025). These pioneering works have made indispensable contributions to our understanding of the biologic basis and clinical practice of different therapies. However, ethical and practical constraints have limited most studies to non-randomized designs with small samples, baseline imbalances, and insufficient follow-up (Dai et al., 2022; Li et al., 2017). In addition, the quantitative analysis of key imaging indicators (such as changes in root canal wall thickness and apical closure rate) in existing studies is insufficient, which cannot provide sufficient evidence-based medical basis for clinicians to choose individualized treatment plans.

Based on the above clinical needs and research gap, this study used a retrospective propensity score-matched cohort study design to longitudinally compare apexification, vital pulpotomy, and revascularization. This study compared the therapeutic efficacy of the three interventions. The following hypotheses were proposed: Revascularization is more advantageous in promoting the growth of root length and root canal wall thickness of immature permanent teeth; Vital pulpotomy and revascularization are superior to conventional apexification in terms of apical closure and overall success rate. Revascularization demonstrated superior periapical lesion healing compared to both vital pulpotomy and apexification. The three groups demonstrated comparable overall complication rates within 12 months postoperatively. These findings provide reliable evidence to inform clinical decision-making and advance treatment optimization for immature permanent teeth.

## 2 Materials and methods

### 2.1 Study design

**FIGURE 1:**
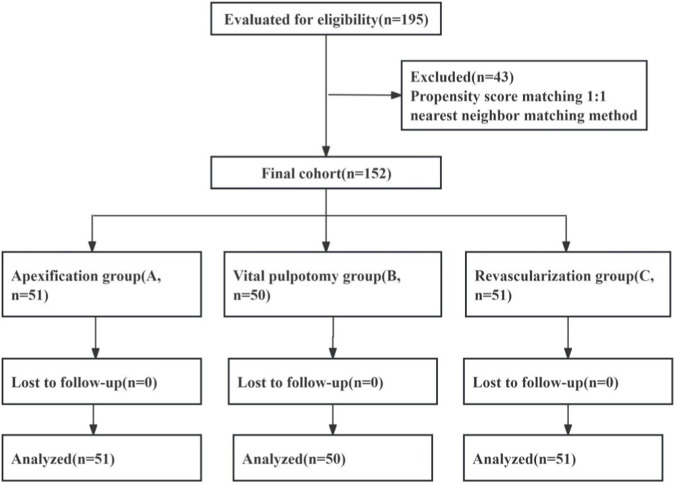
Design flow chart.

In this PSM-based retrospective analysis, 195 patients with immature permanent teeth (January 2022 - December 2024) were selected. Through 1:1 nearest-neighbor matching, three cohorts with balanced baseline characteristics were formed: apexification (n = 51), vital pulpotomy (n = 50), and revascularization (n = 51). The design operation process is shown in Figure 1.

### 2.2 Ethical explanation

This study received ethics approval from the institutional committee of Wuxi Stomatological Hospital with approval number 2022H011, complying with the Declaration of Helsinki and international standards for medical research ethics. As this was a retrospective study using archived clinical data, the ethics committee granted a waiver of informed consent. All study data were anonymized to ensure the privacy and identity of the participants.

### 2.3 Inclusion and exclusion criteria

Inclusion criteria: ①Patients under 18 years old met the definition of young permanent teeth (apical diameter ≥1.0 mm, Nolla stage 7–9) (Nolla, 1960); ②Dental pulp infection or necrosis due to caries, trauma and other reasons should be treated with apexification, vital pulpotomy or revascularization; ③Complete clinical data, including clear periapical radiographs and cone-beam CT images before operation, 6 months and 12 months after operation; ④The patients were in good general health and had no systemic diseases (such as diabetes, immunodeficiency, etc.) that could affect the healing of the pulp tissue.

Exclusion criteria: ①The affected tooth had longitudinal root fracture or severe periodontal bone defect, defined as alveolar bone loss exceeding 1/2 of the root length or presence of vertical bone defects ≥5 mm on radiographic examination (Peng et al., 2018); ②History of systemic diseases or systemic diseases affecting tooth root development; ③Periapical bone destruction was more than one-third of the root length; ④allergy to the drugs used in the treatment; ⑤Loss of follow-up or unclear imaging data during follow-up could not be used for measurement and analysis.

### 2.4 Treatment methods

All the treatment plans of the affected teeth were based on the actual clinical decisions of the patients at the time of consultation. All the operations were performed by experienced endodontists, and their operating procedures were in line with the applicable clinical guidelines and consensus at that time (Duggal et al., 2017). The specific protocols for each group were as follows:

Apexification group: After canal disinfection with 1.5% sodium hypochlorite and saline, the canal was dried. A 4–5 mm apical plug of mineral trioxide aggregate (MTA) was placed using appropriate pluggers, confirmed radiographically. A moist cotton pellet was placed over the MTA to facilitate setting, and the access cavity was temporarily sealed. In a subsequent visit, the remainder of the canal was obturated with gutta-percha and sealer using warm vertical compaction.

Vital pulpotomy group: Following coronal pulp amputation under rubber dam, hemostasis was achieved with moist sterile cotton pellets applied with gentle pressure. The remaining radicular pulp was then covered with a 2–3 mm layer of MTA or Biodentine for anterior teeth to minimize discoloration risk. The material was gently condensed, and the cavity was restored with glass ionomer cement and composite resin.

Revascularization group: After initial disinfection with 1.5% sodium hypochlorite, a triple antibiotic paste (ciprofloxacin, metronidazole, minocycline) was placed into the canal for 2–4 weeks. At the second visit, under rubber dam isolation, the canal was irrigated with 17% EDTA, dried, and bleeding was induced into the canal by over-instrumenting with a sterile hand file beyond the apical foramen. A blood clot was allowed to form up to the level of the cementoenamel junction. MTA (approximately 3 mm) was carefully placed over the clot and allowed to set, followed by a final restoration with glass ionomer cement and composite resin. No adjunctive methods such as platelet-rich plasma (PRP) or leukocyte-platelet-rich fibrin (L-PRF) were used; the revascularization procedure relied solely on the induced blood clot, as recent meta-analyses have shown comparable success rates between blood clot and platelet concentrate techniques (Tsiolaki et al., 2025).

### 2.5 Observation indicators

By querying the hospital electronic medical record system, image archiving and communication system and nursing records, the data of the following observation indicators were retrospectively extracted and collected.

#### 2.5.1 Baseline data

Demographic and clinical data, including age, gender, tooth position, and clinical symptoms (spontaneous pain, pain on percussion, gingival redness/swelling/suppuration, sinus tract formation), were extracted from medical records and nursing records. Preoperative radiographic parameters were assessed using standardized imaging protocols: Root length (mm) and root development stage (Nolla stage) were measured on intraoral periapical radiographs taken with the paralleling technique (XCP, Rinn, United States; 70 kV, 7 mA, 0.2 s); Canal wall thickness at the apical third (mm), apical foramen diameter (mm), presence of periapical radiolucency, and its maximum diameter (mm) were evaluated on cone-beam computed tomography (CBCT) images (3D Accuitomo 170, Morita, Japan; 90 kV, 5 mA, field of view 4 × 4 cm, voxel size 0.125 mm).

All measurements were performed by two calibrated examiners blinded to treatment allocation. Inter-examiner reliability was assessed using intraclass correlation coefficients (ICC >0.85 for all continuous measurements), indicating excellent agreement.

#### 2.5.2 Main outcome measures

Primary outcomes were resolution of clinical signs and symptoms and periapical lesion healing at 12 months postoperatively. Clinical signs and symptoms were assessed based on patient records, including the absence of spontaneous pain, pain on percussion, gingival swelling, suppuration, and sinus tract formation; Periapical lesion healing was evaluated radiographically: In patients with preoperative periapical radiolucency, healing status was categorized as follows: ① Complete healing: complete disappearance of the radiolucent lesion; ② Partial healing: reduction in lesion size ≥50% compared with preoperative dimensions; ③ No healing: reduction <50%, no change, or enlargement of the lesion. The overall healing rate was defined as the proportion of teeth showing either complete or partial healing. Distributions of healing categories were compared among groups (Darwish et al., 2025).

#### 2.5.3 Secondary outcome measures

The changes of root length and root canal wall thickness recorded by the imaging data of the patients before surgery, 6 months and 12 months after surgery were reviewed, and the increased value (postoperative value minus preoperative value) was calculated. The CBCT images of 12 months after surgery were reviewed, and the number of cases with invisible apical foramen or diameter smaller than the resolution limit of the equipment was recorded, and the percentage of these cases in the total cases was calculated (Patel et al., 2026; Xie et al., 2024). Additionally, overall treatment success and complication rates were recorded as composite measures. Treatment success was defined as the absence of clinical symptoms, continued root development or apical closure, no new periapical lesions, and no serious complications (Li et al., 2025; Tong et al., 2022). Complications included root canal calcification, reinfection, and root fracture.

### 2.6 Sample size calculation

The sample size was calculated according to the anticipated change in root canal wall thickness. Referring to relevant literature (Ma et al., 2025), it was reported that the increase in root canal wall thickness in the revascularization group was about 0.28 mm higher than that in the apexification group, and the combined standard deviation was about 0.475 mm. Set α = 0.05 (two-sided), test Power (1-β) = 0.80, using two independent samples t-test, the required sample size for each group was 47 cases calculated by G*Power 3.1 software. Considering the matching efficiency of PSM and the loss rate of about 20%, the final sample size was 152 cases in the apexification group (51 cases), vital pulpotomy group (50 cases) and revascularization group (51 cases), which met the requirements of statistical test.

### 2.7 Statistical analysis

Data analysis was conducted using SPSS 26.0. To reduce selection bias in the retrospective study, PSM was first performed to create a baseline balanced study cohort. A multinomial logistic regression model was used to calculate propensity scores, with treatment group as the dependent variable. The matched variables included age, gender, tooth position, preoperative periapical disease status, apical foramen diameter, root development stage, and key clinical symptoms. 1:1 nearest neighbor matching was used, and the caliper value was set to 0.02. After matching, standardized mean differences (SMD) for all baseline variables were <0.10, indicating balanced baseline characteristics between the groups. Before and after matching, continuous variables were presented as mean ± SD and compared by ANOVA. Categorical variables were expressed as n (%) and analyzed using Pearson’s *χ* ^*2*^ or Fisher’s exact test, as appropriate. The differences of primary and secondary outcomes among the three groups were compared by one-way ANOVA, and Bonferroni correction was used for pairwise comparison. Using *χ* ^*2*^ or Fisher’s exact tests, we derived RR values with 95% CIs. The test level α = 0.05 (two-sided), *P* < 0.05 was considered statistically significant.

## 3 Results

### 3.1 Comparison of baseline data

A total of 195 patients were initially enrolled in the study. In order to reduce confounding bias, the baseline data were balanced by multi-class PSM. Finally, 152 patients were included in the analysis, including 51 patients in the apexification group, 50 patients in the vital pulpotomy group, and 51 patients in the revascularization group. As presented in Table 1, significant intergroup differences in several baseline characteristics were observed prior to matching (*P* < 0.05). Patients in the revascularization group were younger, had a larger apical foramen diameter, a higher proportion of patients with periapical shadow and lesion diameter, an earlier stage of root development, and were more likely to have clinical symptoms that represent acute or acute episodes of chronic periapical periodontitis (e.g., gingival redness, purulent discharge, and fistula formation). In contrast, patients in the vital pulpotomy group showed more features of pulpitis (e.g., spontaneous pain, occlusal pain). After 1:1 PSM matching, the matched cohorts achieved balance on all assessed covariates, including age, gender, tooth position, contralateral teeth, initial root length, initial root canal wall thickness, apical foramen diameter, etiology, periapical lesions, root development stage and clinical symptoms (*P* > 0.05). This indicates that PSM effectively balances the baseline characteristics between the groups, and the three matched groups of patients are sufficiently comparable, which lays the foundation for the fair comparison of subsequent efficacy.

**TABLE 1:** Comparison of patient baseline characteristics.

Indicators	Before PSM				After PSM			
	Apexification group (n = 65)	Vital pulpotomy group (n = 62)	Revascularization group (n = 68)	*P* value	Apexification group (n = 51)	Vital pulpotomy group (n = 50)	Revascularization group (n = 51)	*P* value
Demographics								
Age (years)	11.8 ± 2.1	10.5 ± 1.9	10.2 ± 1.7	<0.001	10.5 ± 1.8	10.7 ± 1.9	10.5 ± 1.9	0.820
Sex (Male, %)	35 (53.8%)	38 (52.8%)	44 (56.4%)	0.912	27 (52.9%)	26 (52.0%)	28 (54.9%)	0.958
Tooth characteristics								
Tooth position (anterior, %)	40 (61.5%)	45 (62.5%)	60 (76.9%)	0.068	33 (64.7%)	32 (64.0%)	35 (68.6%)	0.859
Antagonist tooth present (Yes, %)	58 (89.2%)	65 (90.3%)	68 (87.2%)	0.842	46 (90.2%)	45 (90.0%)	45 (88.2%)	0.954
Preoperative radiographic indicators								
Initial root length (mm)	12.1 ± 1.4	11.9 ± 1.5	11.8 ± 1.6	0.485	11.9 ± 1.5	12.0 ± 1.4	11.9 ± 1.5	0.935
Initial canal wall thickness (mm)	2.04 ± 0.20	2.07 ± 0.18	2.02 ± 0.22	0.352	2.05 ± 0.21	2.06 ± 0.18	2.03 ± 0.22	0.877
Apical foramen diameter (mm)	1.5 ± 0.3	1.6 ± 0.4	2.1 ± 0.5	<0.001	1.7 ± 0.4	1.6 ± 0.3	1.9 ± 0.3	0.197
Preoperative clinical conditions								
Etiology (trauma, %)	35 (53.8%)	40 (55.6%)	35 (44.9%)	0.352	26 (51.0%)	27 (54.0%)	25 (49.0%)	0.874
Periapical radiolucency present (Yes, %)	40 (61.5%)	30 (41.7%)	65 (83.3%)	<0.001	39 (76.4%)	32 (64.0%)	40 (78.4%)	0.210
Periapical lesion diameter (mm)	2.5 ± 1.1	1.8 ± 0.9	3.2 ± 1.3	<0.001	2.6 ± 0.9	2.4 ± 1.0	2.7 ± 1.1	0.115
Root development stage (Nolla’s stage)	8.1 ± 0.5	8.2 ± 0.4	7.9 ± 0.6	0.002	8.0 ± 0.5	8.1 ± 0.5	8.0 ± 0.5	0.948
Clinical symptoms								
Spontaneous pain (Yes, %)	18 (27.7%)	35 (48.6%)	15 (19.2%)	<0.001	16 (31.4%)	17 (34.0%)	15 (29.4%)	0.884
Pain on percussion (Yes, %)	42 (64.6%)	55 (76.4%)	45 (57.7%)	0.047	33 (64.7%)	34 (68.0%)	32 (62.7%)	0.842
Gingival Redness/Swelling/Suppuration (Yes, %)	25 (38.5%)	10 (13.9%)	40 (51.3%)	<0.001	18 (35.3%)	16 (32.0%)	19 (37.3%)	0.832
Sinus tract formation (Yes, %)	15 (23.1%)	2 (2.8%)	20 (25.6%)	<0.001	10 (19.6%)	8 (16.0%)	11 (21.6%)	0.761

PSM, propensity score matching; CBCT, cone-beam computed tomography. Measurements: Root length and Nolla stage were measured on periapical radiographs; canal wall thickness, apical foramen diameter, and periapical lesion dimensions were measured on CBCT, images.

### 3.2 Healing rate of periapical lesions

Preoperatively, periapical lesions were present in 39, 32, and 40 patients in the apexification, vital pulpotomy, and revascularization groups, respectively. At 12 months postoperatively, healing status was categorized as complete healing, partial healing, or no healing (Table 2). The healing rate (complete + partial) differed significantly among groups (χ^2^ = 7.86, *P* = 0.020). Revascularization demonstrated the highest healing rate (95.0%), significantly surpassing both apexification (76.9%) and vital pulpotomy (72.0%) (*P* < 0.05; Table 5). No significant difference was observed between the apexification and vital pulpotomy groups (*P* > 0.05).

**TABLE 2:** Healing of pre-existing periapical lesions at 12 Months.

Group	Pre-op lesions (n)	Complete healing (n)	Partial healing (n)	No healing (n)	Healing rate (%)
Apexification (A)	39	18	12	9	76.9
Vital pulpotomy (B)	32	14	9	9	72.0
Revascularization (C)	40	28	9	3	95.0
*χ* ^*2*^ value	-	-	​	-	7.86
*P* value	-	-	​	-	0.020
Multiple comparisons	-	-	​	-	C>A = B(*P*<0.05)

Healing rate = (Complete + Partial)/Total × 100%. Inter-group comparisons were analyzed by Pearson chi-square test based on binary outcome (healed vs. non-healed). “C > A = B″ denotes that Group C showed significantly higher healing rate than Groups A and B (*P* < 0.05), with no significant difference between Groups A and B.

### 3.3 Root length variation

The three groups had comparable preoperative root length (*P* = 0.935, Table 3). Root length increased in a time-dependent manner after surgery in all groups, but the magnitude of the increase showed significant between-group differences. At 6 and 12 months after operation, the overall comparison between the two groups showed statistical significance (all *P* < 0.05). Post hoc multiple comparisons showed that there was the same trend in the gain value of length at the two time points and 12 months: revascularization group > vital pulpotomy group > apexification group (all *P* < 0.05). Therefore, revascularization shows the best effect in promoting root elongation, followed by vital pulpotomy, while apexification has a relatively limited effect.

**TABLE 3:** Changes in Root Length Across Treatment Groups (x ± s, mm).

Group	n	Preoperative	Postoperative 6 months	Postoperative 12 months	12-month increment
Apexification Group (A)	51	11.9 ± 1.5	12.2 ± 1.4	12.5 ± 1.4	0.6 ± 0.2
Vital pulpotomy Group (B)	50	12.0 ± 1.4	12.8 ± 1.2	13.2 ± 1.2	1.2 ± 0.3
Revascularization Group (C)	51	11.9 ± 1.5	13.0 ± 1.3	13.7 ± 1.3	1.8 ± 0.4
*F* value	-	0.067	5.086	10.927	177.840
*P* value	-	0.935	0.007	<0.001	<0.001
Multiple comparisons	-	-	C > B > A	C > B > A	C > B > A (all *P* < 0.05)

Values represent mean ± SD., Intra-group changes were assessed by paired t-tests; inter-group differences were evaluated using ANOVA, with Bonferroni adjustment. “C > B > A” signifies significantly increasing values across groups (all *P* < 0.05).

### 3.4 Changes in wall thickness of the root canal

The baseline of apical 1/3 canal wall thickness was comparable among the three groups (*P* = 0.877). As shown in Table 4, there was a continuous thickening trend of the root canal wall in all groups after surgery. At the 12-month follow-up, all groups showed significant increases in thickness from baseline (all *P* < 0.01). The revascularization group demonstrated significantly greater improvement (0.35 ± 0.05 mm) compared to both the apexification (0.13 ± 0.06 mm) and vital pulpotomy groups (0.14 ± 0.07 mm). The difference between the two groups at 6 months, 12 months and 12 months after operation was highly statistically significant (all *P* < 0.05). Multiple comparisons confirmed significantly greater root canal wall thickness in the revascularization group versus both other groups at all timepoints (all *P* < 0.05), whereas the apexification and vital pulpotomy groups showed comparable results (*P* > 0.05). The results showed that revascularization was significantly superior to the other two procedures in promoting the thickening of the root canal wall, while apexification and vital pulpotomy had similar thickening effects.

**TABLE 4:** Root canal wall thickness changes in the three groups (x ± s, mm).

Group	n	Preoperative	Postoperative 6 months	Postoperative 12 months	12-month increment
Apexification Group (A)	51	2.05 ± 0.21	2.14 ± 0.19	2.17 ± 0.19	0.13 ± 0.06
Vital pulpotomy Group (B)	50	2.06 ± 0.18	2.16 ± 0.17	2.20 ± 0.17	0.14 ± 0.07
Revascularization Group (C)	51	2.03 ± 0.22	2.23 ± 0.20	2.38 ± 0.22	0.35 ± 0.05
*F* value	-	0.131	3.739	17.534	210.094
*P* value	-	0.877	0.026	<0.001	<0.001
Multiple comparisons	-	-	C>A = B	C>A = B	C>A = B (all *P* < 0.05)

Intra-group comparisons were made using paired t-tests. Inter-group comparisons were analyzed by ANOVA, with Bonferroni correction. “C > A = B” indicates Group C showed significantly greater values than both Groups A and B (*P* < 0.05), while no significant difference was found between Groups A and B (*P* > 0.05).

### 3.5 Apical closure rate

The 12-month apical closure rates differed significantly across groups (χ^2^ = 16.83, *P* < 0.001). Revascularization and vital pulpotomy demonstrated similar efficacy (88.2% vs. 82.0%, *P* > 0.05), as detailed in Table 5. Both groups achieved markedly higher success rates than the apexification group (56.9%, *P* < 0.01). The results showed that vital pulpotomy and revascularization were equally effective in promoting apical closure of young permanent teeth, and both of them were significantly better than traditional apexification.

**TABLE 5:** Apical foramen closure at 12 Months postoperatively.

Group	n	Closed (n)	Not closed (n)	Closure rate (%)
Apexification (A)	51	29	22	56.9
Vital pulpotomy (B)	50	41	9	82.0
Revascularization (C)	51	45	6	88.2
χ^2^ value	-	-	-	16.83
P value	-	-	-	<0.001
Multiple comparisons	-	-	-	C=B > A (*P* < 0.01)

Inter-group comparisons were analyzed by Pearson chi-square test. “B = C > A” denotes no significant difference between Groups B and C, with both superior to Group A (*P* < 0.01).

### 3.6 Success rate of treatment

At 12 months after operation, there was a highly significant difference in the treatment success rate among the three groups based on comprehensive clinical and imaging criteria (*χ* ^*2*^ = 9.36, *P* = 0.009). As shown in Table 6, the results of pairwise comparison between groups showed that the success rate of vital pulpotomy group (group B) and revascularization group (group C) was 92.0% and 88.5%, respectively, and there was no significant difference between the two groups (*P* > 0.05). However, the therapeutic efficacy of both test groups was markedly superior to that of the apexification group (group A, 76.5%), with a statistically significant difference detected (*P* < 0.05). Collectively, these findings demonstrate that vital pulpotomy and revascularization exhibit comparable overall treatment outcomes in the management of immature permanent teeth, and both approaches are notably more effective than conventional apexification therapy.

**TABLE 6:** Overall treatment success rate at 12 months.

Group	n	Success (n)	Failure (n)	Success rate (%)
Apexification (A)	51	39	12	76.5
Vital pulpotomy (B)	50	46	4	92.0
Revascularization (C)	51	45	6	88.5
*χ* ^*2*^ value	-	-	-	9.36
*P* value	-	-	-	0.009
Multiple comparisons	-	-	-	B=C>A (*P* < 0.05)

Inter-group comparisons were analyzed by Pearson chi-square test. “B = C > A” indicates no significant difference between Groups B and C, and both were superior to Group A (*P* < 0.05).

### 3.7 Incidence of complications

The main types of complications were root canal calcification and reinfection, and the incidence of root fracture was very low. As shown in Table 7, there were 9 cases of complications in the apexification group, with a total incidence of 17.6%. The number of complications in the vital pulpotomy group was the least, a total of 4 cases, and the total incidence was 8.0%. Complications occurred in 6 cases in the revascularization group, with a total incidence of 11.8%. Analysis revealed comparable complication rates across the three groups (*χ* ^*2*^ = 1.52, *P* = 0.468). It is suggested that the three treatment methods are consistent in terms of postoperative safety.

**TABLE 7:** Incidence of complications within 12 Months.

Group	n	Canal calcification (n)	Reinfection (n)	Root fracture (n)	Total [n, (%)]
Apexification (A)	51	3	5	1	9 (17.6)
Vital pulpotomy (B)	50	1	2	1	4 (8.0)
Revascularization (C)	51	4	2	0	6 (11.8)
*χ* ^*2*^ value	-	-	-	-	1.52
*P* value	-	-	-	-	0.468

## 4 Discussion

This retrospective cohort study employed strict propensity score matching to compare long-term radiographic outcomes of apexification, vital pulpotomy, and revascularization in treating immature permanent teeth with balanced baseline parameters. Our findings clearly revealed the essential differences in the ability of the three treatments to promote root development and provided important evidence for clinical decision making.

One of the core goals of young permanent teeth treatment is to promote root development to enhance the stability of tooth anatomical structure and reduce the risk of subsequent fracture (Panda et al., 2022). In this study, we found that revascularization was superior to vital pulpotomy and apexification in promoting root length and root canal wall thickening. This result is highly consistent with the conclusion of a recent large meta-analysis by Zhang Q. et al. (2025), which also reported that regenerative pulp therapy (REPs, the core of which is revascularization) has significant advantages over traditional methods in promoting continued root development. The root cause lies in the very different biologic bases of the three therapies. Apexification is essentially an “apical barrier technique” that contributes little to the development of the root itself. Vital pulpotomy continues the physiological development pattern of the root by preserving the healthy root pulp (Hu et al., 2025). The core concept of revascularization is regeneration. The most obvious improvement in root length and canal wall thickness in the revascularization group was the absolute advantage in this study. Our results are further supported by a recent Bayesian network meta-analysis, which, when comparing different biological scaffolds, found that clot based revascularization was comparable to the use of advanced scaffolds such as concentrated growth factors in increasing root length in long-term follow-up (>12 months) (Wang et al., 2024). These results indicate that the regeneration microenvironment created by revascularization itself is the key to promote root development.

Apical maturation stands as a key indicator of therapeutic success in immature permanent teeth, as it mirrors the developmental maturity of the root and the stability of periapical tissues (Yu et al., 2024; Adel and Asgari, 2025). Our study confirms that vital pulpotomy is comparable to revascularization in terms of apical foramen closure rate and comprehensive treatment success rate, and both are significantly higher than apexification. This is consistent with the conclusion of a study conducted by Shanady et al. (2025) comparing human processed dentin matrix (hTDM) and MTA for vital pulpotomy, which also reported a clinical and radiographic success rate of up to 100%. All these findings highlight the irreplaceable role of the preservation of self-viable pulp tissue in maintaining the physiological function and good prognosis of teeth. However, vital pulpotomy was superior to apexification but inferior to revascularization in promoting the thickening of the root canal wall. This may imply that the odontogenic potential of the retained pulp after partial infection and surgical trauma is somewhat limited, and its ability to drive root thickening is not comparable to that of a theoretically “*de novo*” regenerative system. In terms of periapical lesion healing, revascularization was significantly better than the other two groups. This result is highly consistent with the reports in the literature. Arslan et al. (Arslan et al., 2019) found that the healing rate of periapical lesions after regenerative pulp therapy could reach 92.3%, which was significantly higher than 80% of traditional root canal therapy. This is closely related to the ability of this technique to reconstruct the blood supply and defense system in the root canal. The regenerated tissue not only promotes the deposition of hard tissue, but also may reconstruct the immune microenvironment in the root canal, thereby eliminating residual infection more effectively and promoting the repair and regeneration of periapical tissues. However, the healing of periapical lesions by apexification and vital pulpotomy relies more on traditional root canal disinfection and strict filling, and their active immune repair ability is relatively weak (Sun et al., 2025; Ramezani et al., 2019; Costa et al., 2013). In this study, the number of patients with periapical lesions before operation in the revascularization group was more than that in the other two groups, but the healing rate was still high, which further confirms the value of revascularization in the treatment of periapical lesions. These findings suggest that revascularization should be considered as the preferred treatment for immature permanent teeth with periapical disease, especially those with large extent of disease, which can achieve infection control and tissue repair more efficiently and improve the prognosis of the affected teeth.

The overall incidence of complications did not differ significantly among the three groups, indicating that revascularization, as a more complex treatment option, does not carry higher short-term risks. The risk of reinfection after apexification is relatively high, which may be related to the incomplete apical barrier formation or coronal microleakage. Although the difference was not statistically significant, it is noteworthy that the revascularization group exhibited a numerically higher occurrence of root canal calcification compared to the other groups. This observation aligns with recent study reporting that intracanal calcification is a common finding following regenerative endodontic procedures (Song et al., 2025). From a biological perspective, this calcification may represent a “double-edged sword”: on one hand, it reflects the successful revascularization and revitalization of the pulp-dentin complex, indicating active tissue regeneration and hard tissue deposition by newly formed odontoblast-like cells (Huang et al., 2008); on the other hand, progressive calcification could potentially complicate future endodontic retreatment if necessary, and may be associated with crown discoloration, particularly when minocycline-containing triple antibiotic paste is used (Kahler et al., 2016). Clinically, this underscores the importance of long-term follow-up for patients undergoing revascularization, as well as the need to balance the regenerative benefits against the potential for calcific metamorphosis. Future research should explore strategies to modulate the extent of calcification, such as optimizing disinfection protocols or using alternative antibiotic combinations, to maximize regenerative outcomes while minimizing undesirable obliteration (Nagata et al., 2014). In clinical application, individualized treatment should be selected according to the condition of the tooth and the patient’s condition. For the cases of root hypoplasia, severe apical lesions, and complete pulp necrosis, revascularization is preferred to maximize root development and lesion healing. For cases with partial pulp necrosis but without infected root pulp (such as coronal pulp necrosis after trauma), vital pulpotomy can be selected to take into account the efficacy and operation simplicity. Apexification is still a reliable alternative for young children, patients who are unable to cooperate with revascularization procedures, or patients with complicated anatomical structures (such as excessive tortuosity of root canals) (Shah et al., 2024; Aksoy and Kocak, 2025). In addition, standardized operation (such as thorough root canal preparation and good sealing) and long-term follow-up should be emphasized to reduce the risk of complications and ensure the therapeutic effect.

## 5 Conclusion

In conclusion, the longitudinal radiographic evaluation of this study demonstrated clear hierarchies of efficacy among revascularization, vital pulpotomy and apexification in the treatment of immature permanent teeth. Revascularization has significant advantages in promoting root development and periapical tissue regeneration. Vital pulpotomy is effective in maintaining pulp vitality and achieving apical closure. Apexification is a reliable terminal treatment. Clinicians should carefully select the best individualized treatment plan according to the specific pulp vitality, infection degree and root development stage of the affected tooth. Of course, there are some limitations of this study. First, although the retrospective design controlled the confounding bias as much as possible through PSM, it could not completely avoid the influence of potential unknown confounding factors. Second, as this research was conducted at a single institution, the extrapolation of the conclusions needs to be verified by more research centers. Third, although the follow-up period of 12 months can effectively evaluate the root development trend, an extended follow-up timeframe is crucial for monitoring the long-term stability of therapeutic outcomes and the incidence of delayed complications, such as crown discoloration and complete root canal calcification. Fourth, this study did not assess pulp sensibility testing or histological outcomes, which are considered tertiary endpoints in regenerative endodontic therapy. Future studies incorporating these measures could provide deeper insights into the true regenerative potential of each treatment modality. Fifth, as a retrospective study, pain assessment was limited to binary recording (present/absent) from medical records, rather than using validated pain scales such as the Visual Analogue Scale (VAS) or FLACC scale. Prospective studies should incorporate standardized pain assessment tools to enable more precise evaluation of treatment outcomes. Prospective multi-center cohort investigations incorporating an extended follow-up duration ought to be conducted in subsequent research to further validate the long-term therapeutic efficacy and safety profiles of the three treatment modalities. At the same time, the optimization of biological materials and the precise definition of indications can be explored to provide more efficient and accurate evidence-based basis for the treatment of immature permanent teeth.

## Data Availability

The original contributions presented in the study are included in the article/supplementary material, further inquiries can be directed to the corresponding author.
